# Ventral Hippocampal Input to Infralimbic Cortex Is Necessary for the Therapeutic-Like Effects of Extinction in Stressed Rats

**DOI:** 10.1093/ijnp/pyad043

**Published:** 2023-07-22

**Authors:** Denisse Paredes, David A Morilak

**Affiliations:** Department of Pharmacology and Center for Biomedical Neuroscience, University of Texas Health Science Center at San Antonio, San Antonio, TX, USA; Department of Pharmacology and Center for Biomedical Neuroscience, University of Texas Health Science Center at San Antonio, San Antonio, TX, USA; South Texas Veterans Health Care System, San Antonio, TX

**Keywords:** Chronic stress, fear extinction, infralimbic cortex, ventral hippocampus

## Abstract

**Background:**

Posttraumatic stress disorder is characterized by deficits in cognitive flexibility related to dysfunction of the medial prefrontal cortex (mPFC). Exposure therapy can effectively reverse these deficits. Fear extinction in rodents bears similarity to exposure therapy. Extinction reverses chronic stress–induced deficits in cognitive flexibility on the attentional set-shifting test (AST), an mPFC-mediated process. This therapeutic effect requires activity of pyramidal neurons and brain derived neurotrophic factor (BDNF) signaling in infralimbic cortex (IL). However, the circuit mechanisms governing BDNF-mediated plasticity initiated by extinction in IL are unknown. The ventral hippocampus (vHipp) plays a role in regulating IL activity during extinction, and plasticity in vHipp is necessary for extinction memory consolidation. Therefore, we investigated the role of vHipp input to IL in the effects of extinction in reversing stress-induced cognitive deficits.

**Methods:**

vHipp input to IL was silenced using a Gi-Designer Receptors Exclusively Activated by Designer Drugs (DREADD) via local infusion of clozapine-N-oxide (CNO) into IL before extinction. A day later, rats were tested on AST. In a separate experiment, we tested whether vHipp input to the IL induces BDNF signaling to exert therapeutic effects. We activated the vHipp using a Gq-DREADD, and injected an anti-BDNF neutralizing antibody into IL. Rats were tested on the AST 24 hours later.

**Results:**

Silencing the vHipp input to IL prevented the beneficial effects of extinction in reversing stress-induced cognitive deficits. Activating vHipp input to IL in the absence of extinction was sufficient to reverse stress-induced deficits in set-shifting. The beneficial effects were blocked by local infusion of a neutralizing anti-BDNF antibody into IL.

**Conclusions:**

vHipp-driven BDNF signaling in IL is critical for extinction to counteract the deleterious cognitive effects of chronic stress.

**Significance Statement**

In this work, we report the importance of ventral hippocampal input to the infralimbic cortex for the therapeutic-like effects of fear extinction learning in stressed rats. We demonstrate that BDNF plays a pivotal role in inducing plasticity via the ventral hippocampal-IL circuit in stressed animals. Such findings elucidate a potential therapeutic target for the treatment of posttraumatic stress disorder.

## INTRODUCTION

Posttraumatic stress disorder (PTSD) and major depressive disorder (MDD) are debilitating illnesses that affect millions every year. Such disorders are characterized by behavioral disruptions associated with dysfunction of the medial prefrontal cortex (mPFC), such as maladaptive coping and cognitive deficits (Murrough et al., 2011; Girotti et al., 2017). Cognitive flexibility deficits are a symptom dimension shared across several psychiatric disorders, and poor cognitive flexibility is associated with increased symptom severity in PTSD. Cognitive flexibility is defined as the ability to modify previously learned associations or responses based on changes in the environment. Chronic stress negatively impacts the function of the mPFC, a region that mediates set shifting, a form of cognitive flexibility (Birrell and Brown, 2000; Liston et al., 2006).

Available pharmacological treatments for PTSD and MDD are ineffective for a large portion of patients and often fail to address cognitive symptoms (Nutt et al., 2010). Behavioral therapies, such as exposure therapy, can be effective in some patients for whom pharmacotherapy is ineffective and can relieve cognitive symptoms of MDD and PTSD. Exposure therapy engages brain regions dysregulated in these disorders (i.e., medial prefrontal cortex, hippocampus, amygdala) (Andero and Ressler, 2012). Unfortunately, only a fraction of patients reach full remission following exposure therapy (de Kleine et al., 2013). Thus, identifying the mechanisms underlying exposure therapy could potentially inform the development of more efficacious treatments targeting such mechanisms.

Fear extinction in rodents engages circuits similar to those activated by exposure therapy (Andero and Ressler, 2012; Paredes and Morilak, 2019). Fear extinction induces plasticity in brain regions susceptible to the effects of stress, such as mPFC and the hippocampus (Sierra-Mercado et al., 2011). mPFC hypoactivity is reported in patients with stress-related psychiatric disorders (Rogers et al., 2004). In rodents, chronic stress attenuates mPFC responsivity to afferent input, and extinction treatment restores responsivity to nonstressed levels (Fucich et al., 2018). As such, extinction may restore healthy function of the mPFC that has been diminished by chronic stress. We have previously demonstrated the effects of extinction used as an intervention to reverse stress-induced behavioral phenotypes. Extinction reverses stress-induced deficits on set shifting and restores active coping on the shock-probe test (Fucich et al., 2016). Similarly, rats exposed to a learned safety model exhibit reduced anxiety on the elevated plus maze and reduced immobility in the forced swim test (Pollak et al., 2008).

We have shown that chemogenetically silencing pyramidal neurons in infralimbic cortex (IL) during extinction attenuates the effects of extinction in reversing stress-induced deficits on set-shifting performance and active coping (Fucich et al., 2018). Further, chemogenetically activating IL pyramidal neurons, in lieu of extinction treatment, reverses stress-induced deficits in set shifting and coping style choice (Fucich et al., 2018). In addition, *de novo* protein synthesis in the IL is necessary for the effects of extinction to reverse stress-induced deficits in set shifting (Fucich et al., 2016). Together, these findings suggest a role for extinction-induced plasticity in the IL in mediating the beneficial effects of extinction in stressed animals.

We recently demonstrated the necessity of brain derived neurotrophic factor (BDNF) signaling in the IL for the therapeutic effects of extinction on set shifting in stressed animals (Paredes et al., 2021). BDNF plays a vital role in memory acquisition and consolidation (Tyler et al., 2002). In both rodents and humans, the BDNF Val66Met polymorphism, which is associated with reduced BDNF secretion, impairs fear extinction (Egan et al., 2003). Extinction induces phosphorylation of the BDNF receptor Tropomyosin receptor kinase B (TrkB) at tyrosine residue Y515 in the IL. Sequestering BDNF in the IL at the time of extinction prevented the reversal of stress-induced deficits in set-shifting performance tested 24 hours later, and exogenous administration of BDNF in the IL, in lieu of extinction, was sufficient to mimic the effects of extinction on set-shifting performance (Paredes et al., 2021). These observations point to BDNF signaling as a crucial component of extinction-induced plasticity in the IL to reverse cognitive impairments caused by stress. However, the mechanisms driving the initiation of BDNF signaling during extinction in stressed animals are unknown.

Specifically, it is unknown if extinction-induced BDNF signaling in the IL is dependent on afferent input from another region, such as the ventral hippocampus (vHipp). Activity-dependent release of BDNF in hippocampal-prefrontal circuits regulate fear extinction learning and behavioral perseverance (Sakata et al., 2013). vHipp neurons projecting to the IL, but not to the amygdala, exhibit increased BDNF expression following extinction of avoidance learning (Rosas-Vidal et al., 2018), and reducing BDNF production in these neurons impairs extinction of avoidance. Indeed, exogenous BDNF administration in the vHipp mimics fear extinction in fear conditioned animals, and blocking BDNF in the IL prevents this effect (Peters et al., 2010). Therefore, we hypothesized that vHipp neurons projecting to the IL may be responsible for BDNF-associated plasticity necessary for extinction to reverse stress-induced cognitive deficits in set shifting. In these experiments, we investigated the necessity of ventral hippocampal input to the IL for the effects of extinction in stressed animals. We further tested whether activating ventral hippocampal input to the IL was sufficient to reverse stress-induced set shifting impairments and whether this effect was dependent upon BDNF signaling in the IL.

## MATERIALS AND METHODS

### Animals

A total of 109 male and female rats (Envigo, 225–249 g) were housed on a 12-hour light/dark cycle. After 1-week acclimation, rats were single housed. Experiments took place during the lights-on portion of the cycle. All procedures followed National Institutes of Health guidelines and were approved by the UTHSA Institutional Animal Care and Use Committee. Group sizes were determined by power analysis using estimates of effect size from previous data. Males and females were included in all groups. As per National Institutes of Health mandate, after the primary analyses, secondary analyses were conducted separately after disaggregating by sex, and results were shown separately for males and females. These experiments, however, were not explicitly powered to study sex differences.

### Fear Conditioning and Extinction

Two days before beginning chronic unpredictable stress (CUS), rats were habituated to 2 contexts in sound-attenuating chambers for 15 minutes each, as previously described (Paredes et al., 2021). Context A consisted of a metal chamber (30.5 × 25.4 × 30.5 cm; Coulbourn Instruments model H10-11RC-TC-Sf) with square walls and a grid floor attached to a shock box (model H13-15). Context B, which was not associated with shock, had a white and green smooth vinyl floor and circular pink and white vinyl walls.

#### Day 0: Fear Conditioning

Rats received fear conditioning or tone control treatment in Context A. Fear conditioning consisted of 4 pairings of a tone (10 kHz, 75 dB, 20 seconds) coterminus with a footshock (0.8 mA, 0.5 seconds), with an inter-trial interval of 120 seconds. Freezing during each tone was quantified using FreezeView (ActiMetrics #ACT-100, Coulbourn Instruments). Tone control rats did not receive footshocks during tone exposures.

#### Day 17: Fear Extinction

All rats received fear extinction in Context B, consisting of 16 tone trials with no shock, with average inter-trial interval of 120 seconds.

#### Chronic Unpredictable Stress

CUS was conducted as previously described (Fucich et al., 2018; Bulin et al., 2020). In male rats, CUS consisted of 14 days of various acute psychogenic stressors (30-minute restraint, 1-hour shake, 10-minute tail pinch, 24-hour wet bedding, 15-minute mild footshock, overnight lights on). As reported previously, 14 days of CUS is not sufficient to induce set-shifting deficits in females, so 21-day CUS was used for females. This induces the same deficit observed in males after 14 days CUS (Bulin et al., 2020). Further, when these procedures produced similar stress-compromised starting points in males and females, we have shown comparable effects of extinction (Paredes et al., 2021, 2022).

#### Attentional Set-Shifting Test (AST)

The AST was used to measure cognitive flexibility on the extradimensional set-shifting task as previously described (Fucich et al., 2016). Rats were food restricted (66% of average daily intake) for 7 days before behavioral testing. Rats were trained and tested in a white plastic arena containing a start gate at one end and 2 terracotta pots separated by a Plexiglas wall at the other end. The AST entails 3 days (habituation, training, and testing), with an extra day inserted between training and testing to allow for extinction treatment. Behavioral procedures began 1 day after the end of CUS (day 15 for males, day 22 for females; see timelines in Figures 2A and 3A):

1. **Habituation day:** Rats were trained to dig in pots filled with sawdust to retrieve a food reward, one-half of a Honey Nut Cheerio (General Mills Cereals, Minneapolis, MN, USA).
2. **Training day:** Rats were trained to locate the reward in 1 of 2 pots by discriminating cues in 2 stimulus dimensions: the odor applied to the rim of the pot or the texture of the digging medium that filled the pot.
3. **Treatment day:** Rats received bilateral microinjections and extinction or tone control treatment.
4. **Testing day:** Rats were tested on a series of discrimination stages in which a criterion of 6 correct consecutive trials were required to proceed to the next stage (Table 1). Rats were tested using either medium or odor as the relevant dimension in the early discrimination stages (i.e., simple discrimination, complex discrimination, reversal learning, intradimensional shift, second reversal), leading to formation of a cognitive set. In the extra-dimensional (ED) set-shifting task, the previously relevant dimension (e.g., odor), was irrelevant, whereas the previously irrelevant dimension (medium) now indicated the location of the Cheerio.

**Table 1.** Attentional set-shifting test (AST), sequence of discrimination tasks comprising the AST

Discrimination Stage	Dimensions		Example combinations	
	Relevant	Irrelevant	(+)	(-)
Simple	Odor		Clove/sawdust	Nutmeg/sawdust
Compound	Odor	Medium	Clove/raffia	Nutmeg/yarn
			Clove/yarn	Nutmeg/raffia
Reversal 1	Odor	Medium	Nutmeg/raffia	Clove/yarn
			Nutmeg/yarn	Clove/raffia
Intradimensional shift	Odor	Medium	Rosemary/wood balls	Cinnamon/plastic beads
			Rosemary/plastic beads	Cinnamon/wood balls
Reversal 2	Odor	Medium	Cinnamon/plastic beads	Rosemary/wood balls
			Cinnamon/wood balls	Rosemary/plastic beads
Extradimensional shift (ED)	Medium	Odor	Velvet/citronella	Crepe/thyme
			Velvet/thyme	Crepe/citronella

All data shown and analyzed are from the extra dimensional (ED) set shifting task.

#### Extinction With Viral Designer Receptors Exclusively Activated by Designer Drugs (DREADD) Delivery and CNO Microinjections

To express an inhibitory DREADD in pyramidal cells in the vHipp, rats underwent stereotaxic surgery for bilateral viral microinjections (0.5 μL per side) of either AAV5-CaMKIIa-hM4D(Gi)-mCherry or the control AAV5-CaMKIIa-EGFP (Addgene) at a rate of 0.1 μL/min into the ventral hippocampus (AP −5.3, ML +/−5.2, DV −7.5; Paxinos and Watson, 2007 ). After injection, the injectors stayed in place for 5 minutes before withdrawal. Rats were then implanted with microinjection guide cannulae (Plastics One, Roanoke, VA, USA) positioned 1 mm above the IL (AP +2.9, ML −3.1, DV −3.8) at a 30° angle to avoid the prelimbic cortex. Rats recovered from surgery for 7 days. On day 0, they received fear conditioning or tone control exposure, and on days 1–14 (1–21 for females) they underwent CUS. On the 2 days following CUS, rats underwent habituation and training for the AST. They then received a bilateral microinjection of CNO immediately before extinction on day 17 for males (day 24 for females). Infusion cannulae extending 1 mm beyond the guide cannulae were inserted into the IL. In experiment 1, rats received bilateral microinjections of CNO (300 μM, 0.75 μL per side at a rate of 0.5 μL/min) into the IL. This dose and method of delivery was taken from a previous study (Donegan et al., 2019). The injectors remained in place for 2 minutes to allow for diffusion, after which rats underwent extinction. After behavioral experiments, brains were checked for cannulae placement in the IL and for viral expression in the ventral hippocampus.

#### Microinjections of CNO and Anti-BDNF in Place of Extinction

Rats received bilateral viral delivery (0.5 μL per side) of either control AAV5-CaMKIIa-EGFP or the excitatory DREADD, AAV5-CaMKIIa-hM3D(Gq)-mCherry into the ventral hippocampus (AP −5.3, ML ±5.2, DV −7.5) as above. Subsequently, rats were implanted with microinjection guide cannulae targeting vHipp terminals in the IL, as above. Rats recovered for 7 days after surgery, then underwent CUS. On the third day after CUS (1 day before testing), rats received bilateral microinjections of CNO into the IL (300 μM, 0.75 μL per side) in lieu of extinction and subsequently received bilateral microinjections of either sheep control IgG or sheep anti-BDNF (EMD Millipore, Billerica, MA,USA; 0.5 µg/0.5 µL per side at a rate of 0.25 μL/min). This dose of anti-BDNF previously was shown to block BDNF signaling (Alonso et al., 2002). The injector remained in place for 2 minutes for diffusion before withdrawal. Rats were returned to housing and tested on the AST drug free the following day.

#### Western Blotting and Tissue Collection

In previous experiments, we observed phosphorylation of TrkB 30 minutes after extinction (Paredes et al., 2021). Therefore, a separate cohort of rats was killed via rapid decapitation 30 minutes after the end of fear extinction for tissue collection. The IL cortex was dissected from a 2-mm coronal slab as previously described (Paredes et al., 2021). The tissue was stored at −80°C until further processing. Western blots were conducted as previously described (Paredes et al., 2021). After transfer, membranes were incubated in a rabbit antibody against pTrkB Y515 (1:20 000, Abcam) then incubated in anti-rabbit secondary antibody (1:5000, Cell Signaling) and detected using ECL Prime (GE Healthcare, Little Chalfont, UK). Membranes were then stripped and reprobed in a goat antibody against TrkB (1:1000, Neuromics) and secondary anti-goat antibody (1:20 000, Cell Signaling).

## RESULTS

### Inhibiting Ventral Hippocampal Terminals in IL Decreases Phosphorylation of TrkB After Extinction

**Figure 1.**
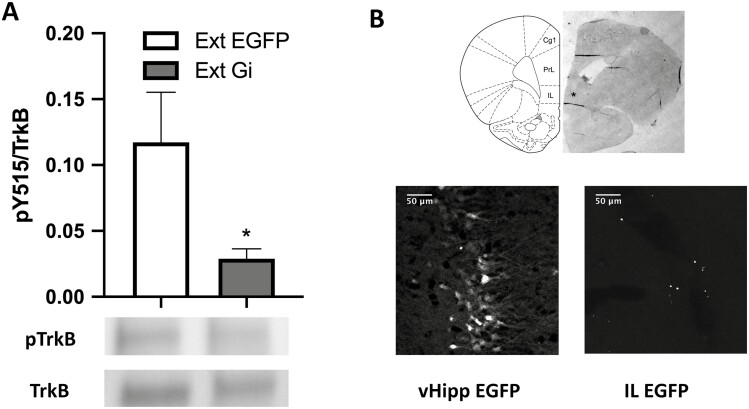
Chemogenetically silencing ventral hippocampal (vHipp) terminals in the infralimbic cortex(IL) decreases TrkB phosphorylation in the IL after extinction. (A) Inhibiting vHipp terminals in the IL attenuated the phosphorylation of TrkB in the IL after extinction (*P < .05). Bars represent SEM. (B) Top: Histological representation of a guide cannula track targeting IL cortex, indicated by the asterisk marking the clozapine-N-oxide injection site located 1 mm beyond the cannula tip (diagram from Paxinos and Watson, 2007). Bottom left: Expression of virus in cell bodies in the vHipp, indicated by the EGFP reporter. Bottom right: Expression of virus in ventral hippocampal terminals in the IL, indicated by the EGFP reporter. Abbreviations: Cg1, cingulate cortex, area 1; IL, infralimbic cortex; PrL, prelimbic cortex. Scale bar = 50 μm.

To assess whether inhibiting ventral hippocampal terminals during extinction affects the phosphorylation of the receptor TrkB at the Y515 residue, 9 males (4–5 per group) and 10 female rats (4–6 per group) were used in 2 groups: (1) a control group with the control EGFP construct injected in the vHipp (Ext-EGFP); and (2) a group injected with the Gi DREADD in vHipp (Ext-Gi). Inhibiting ventral hippocampal input to the infralimbic cortex during extinction significantly attenuated the induction of phosphorylated TrkB in the IL (unpaired *t* test, t_16_ = 2.140, *P* = .0328; Figure 1A). Figure 1B shows an AAV-DREADD microinjection site in vHipp and terminal labeling in IL visualized by GFP reporter expression. CNO microinjection sites were also localized to IL by histological examination (Figure 1B).

### Ventral Hippocampal Input to IL Is Necessary for Effects of Extinction

**Figure 2.**
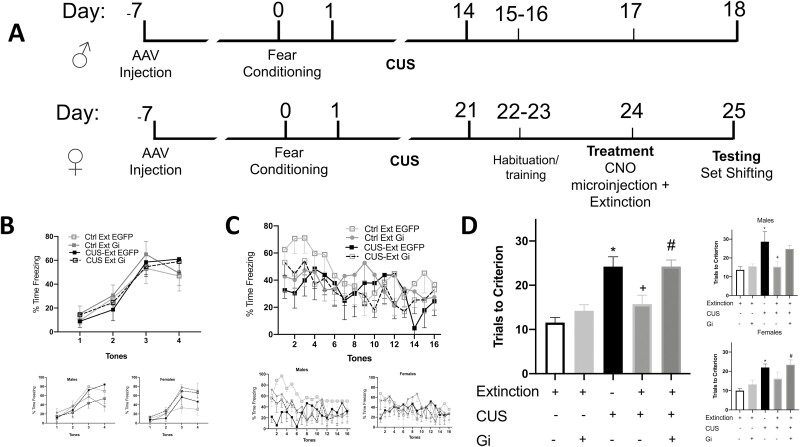
Ventral hippocampal input to the infralimbic cortex (IL) is necessary for the effects of extinction after chronic stress. (A) Experimental timelines for behavioral experiments in male (14-day CUS) and female rats (21-day CUS). (B) Fear conditioning did not differ between groups (i.e., before stress exposure). Insets show male and female data separately (n = 3–6 per group for both sexes). (C) Freezing during fear extinction also did not differ between groups. Insets show male and female data separately (n = 3–6 per group for both sexes). Freezing behavior for some rats could not be scored due to technical issues (the rats managed to hide behind the mat used to form the circular enclosure while freezing during some of the tone presentations). (D) Chemogenetically silencing ventral hippocampal terminals in the IL during extinction blocked the therapeutic effects of extinction. Chronic stress induced a cognitive deficit in the extradimensional set shifting task (*P < .001). This effect was reversed by extinction (^+^*P* < .001). Inhibiting ventral hippocampal input in the IL during extinction blocked the effects of extinction (^#^*P* < .001) (n = 10–13 per group). Data disaggregated by sex are shown separately in the insets (males top, n = 3–8 per group; females bottom, n = 3–6 per group). Bars represent SEM.

To test if inhibiting ventral hippocampal terminals in the IL prevented the effects of extinction in reversing stress-induced deficits in set shifting, 25 males (3–8 per group) and 23 females (3–6 per group) were used in 5 groups. The groups were defined by virus injection, stress condition, and extinction treatment: (1) control group with EGFP injected in the vHipp (no stress/extinction/EGFP); (2) control group with Gi DREADD in the vHipp (no stress/extinction/Gi); (3) stress group, with CUS + tone control and EGFP in the vHipp (CUS/tones/EGFP); (4) stress plus extinction group, with CUS + extinction and EGFP in the vHipp (CUS/extinction/EGFP); and (5) a group to test the necessity of vHipp terminal activity in IL during extinction after stress (CUS/extinction/Gi). Microinjections were administered 24 hours before testing, immediately before extinction. Within-session freezing during extinction did not differ between fear-conditioned groups (F_3,27 _=_ _2.088, *P* = .1252). ANOVA was used to compare performance on the ED set-shifting test between groups. In the primary analysis, ANOVA revealed a significant group effect (F_4,43 _=_ _12.40, *P* < .0001; Figure 2). Pairwise comparisons using the Holm-Sidak test showed stress impaired set-shifting performance (CUS/tones/EGFP compared with no stress/extinction/EGFP, *P* = .0002). Inhibiting vHipp terminals in nonstressed animals at the time of extinction had no behavioral effect on the AST tested 24 hours later (no stress/extinction/EGFP vs no stress/extinction/Gi, *P* = .6901). Consistent with our previous work, extinction reversed stress-induced deficits in set shifting (CUS/tones/EGFP compared with CUS/extinction/EGFP, *P* = .00113). Silencing vHipp terminals in the IL at the time of extinction blocked the effects of extinction on set shifting after CUS (CUS/Extinction/EGFP compared with CUS/extinction Gi, *P* = .0006). Thus, chemogenetically inhibiting vHipp terminals in the IL during extinction blocked the therapeutic effects of extinction in stressed animals. Analyzed separately after disaggregating by sex, males also showed a significant group effect (F_4,19 _=_ _5.588, *P* = .0038; Figure 2D top inset). Pairwise comparisons using the Holm-Sidak test showed stress impaired set shifting (CUS/tones/EGFP compared with no stress/extinction/EGFP, *P *= .0419), and extinction reversed the effects of stress (CUS/tones/EGFP vs CUS/extinction/EGFP, *P* = .0385). Inhibiting vHipp terminals in the IL during extinction blocked the effects of extinction, although the effect was marginally significant due to reduced power after disaggregation (CUS/extinction/EGFP vs CUS/extinction/Gi, *P* = .0532). Similarly, females showed a significant group effect (F_4,19 _=_ _7.475, *P* = .0009; Figure 2D bottom inset). In females, stress induced a cognitive deficit (CUS/tones/EGFP vs no stress/extinction/EGFP, *P* = .0082), which was reversed by extinction, although this effect was again marginally significant due to reduced power after disaggregation (CUS/tones/EGFP vs CUS/extinction/EGFP, *P* = .0585). Inhibiting vHipp terminals in the IL blocked the therapeutic effects of extinction (CUS/extinction/EGFP vs CUS/extinction/Gi, *P* = .0394).

### Activating Ventral Hippocampal Input to IL Reverses Stress Effects on Set-Shifting and is BDNF-Dependent

**Figure 3.**
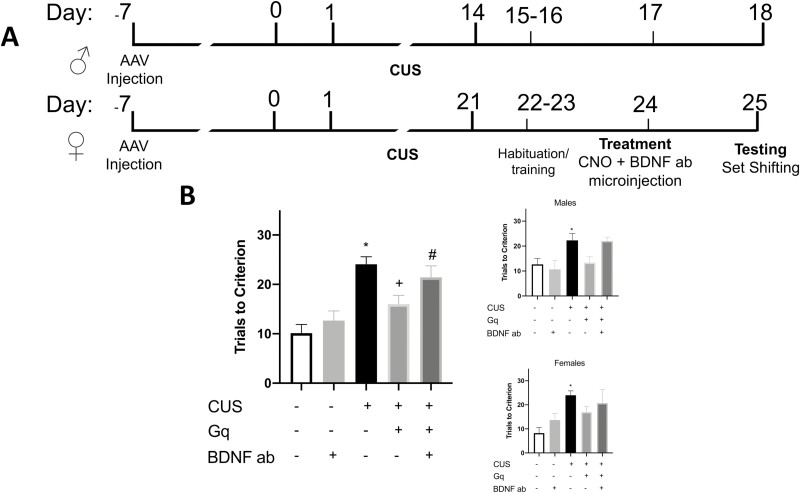
Chemogenetic activation of ventral hippocampal terminals in the infralimbic cortex (IL) reverses stress-induced deficits in set shifting, and this effect is dependent on Brain-Derived Neurotrophic Factor(BDNF) signaling. (A) Experimental timelines for male (14-day CUS) and female rats (21-day CUS). (B) Chemogenetic activation of ventral hippocampal terminals in the IL is sufficient to reverse stress induced deficits in set-shifting performance. Stress induced a deficit in set shifting (*P < .05) that was reversed by chemogenetic activation of ventral hippocampal terminals in the IL (^+^*P* < .05). Blocking BDNF with a neutralizing antibody in the IL prevented the beneficial effects of chemogenetic activation (^#^*P* < .05) (n = 7–11 per group). Data disaggregated by sex are shown separately in the insets (males top, (n = 3–6 per group); females bottom, (n = 3–8 per group). Bars represent SEM.

We next investigated whether (1) chemogenetically activating vHipp input to the IL is sufficient to reverse stress-induced deficits in set shifting, and (2) if these effects are dependent on BDNF signaling in the IL. To address these questions, 19 males (3–6 per group) and 23 females (3–8 per group) were used in 5 groups. The groups were defined by virus injections in the vHipp, IgG injection in the IL, and CUS: (1) no stress with EGFP in vHipp and control sheep IgG in IL (no stress/EGFP/IgG); (2) no stress with EGFP in vHipp and anti-BDNF in IL (no stress/EGFP/anti-BDNF); (3) CUS with EGFP in the vHipp and control sheep IgG; (4) CUS with Gq in the vHipp and sheep IgG injected in IL, to test the sufficiency of activating vHipp terminals to reverse stress-induced set shifting deficits (CUS/Gq/IgG); and (5) a group to test the necessity of BDNF signaling in the IL for the effects of activating vHipp terminals (CUS/Gq/anti-BDNF). ANOVA revealed a significant effect of group on performance in the ED task (F_4,37 _=_ _9.812, *P* = .0001). Pairwise comparisons using the Holm Sidak test showed a significant stress effect (CUS/EGFP/IgG vs no stress/EGFP/IgG, *P* = .0003). Chemogenetic activation of vHipp terminals in the IL reversed the effects of stress (CUS/Gq/IgG vs CUS/EGFP/IgG, *P* = .0366). Infusion of anti-BDNF antibody into the IL prevented the beneficial effects of activating vHipp terminals (no stress/EGFP/IgG vs CUS/Gq/anti-BDNF, *P* = .0057; Figure 3B). In sum, activation of vHipp terminals with Gq was sufficient to reverse the effects of stress on set-shifting performance, and BDNF signaling in the IL was necessary for these effects. Similar patterns of effect were observed in the secondary analyses after disaggregating by sex, although power was not sufficient to achieve significance in all comparisons. In males, set-shifting performance was significantly different between groups (F_4,14 _=_ _5.707, *P* = .0061). Stress induced a set-shifting deficit (CUS/EGFP/IgG vs no stress/EGFP/IgG, *P* = .0485), which was nonsignificantly reversed by Gq activation (CUS/Gq/IgG vs CUS/EGFP/IgG, *P* = .0625). Likewise in females, set-shifting performance significantly differed between groups (F_4,18 _=_ _4.339, *P* = .0125). In females, stress induced a deficit on set-shifting performance (NS/EGFP/IgG vs CUS/EGFP/IgG, *P* = .0102), which was also nonsignificantly reversed by Gq activation.

## DISCUSSION

These results demonstrate the necessity of glutamatergic vHipp input to the IL for extinction to reverse stress-induced deficits in set-shifting performance, consistent with literature highlighting the importance of plasticity in these regions for successful extinction memory recall (Sierra-Mercado et al., 2011; Sakata et al., 2013). Although the focus of our studies was not to discern whether silencing vHipp input to the IL affects extinction memory itself, several studies have consistently shown that inactivating or hindering plasticity in the vHipp impairs extinction recall (Sierra-Mercado et al., 2011; Park et al., 2020). Our assumption is not that inhibiting IL terminals will affect extinction but rather that it will affect the downstream consequences of plasticity induced by extinction. In previous work, we found that BDNF signaling in the IL is necessary to reverse stress-induced deficits on set shifting. We also found that exogenous BDNF administration in the IL is sufficient to reverse stress induced deficits on set shifting, recapitulating the beneficial effects of extinction in stressed animals. Further, we identified molecular signaling pathways (e.g., Erk signaling) induced by extinction learning in the IL that are necessary to reverse stress-induced cognitive deficits. Thus, our studies suggest that extinction induces long-lasting molecular processes induced by BDNF in the IL and are necessary to reverse the cognitive effects of stress. Our data suggest that the vHipp is not only encoding contextual information during extinction (Hobin et al., 2006), but it is also modulating IL plasticity associated with reversing the detrimental effects of stress on other cognitive processes mediated in the mPFC. Chronic stress contributes to loss of dendritic processes in both the vHipp and mPFC (Sapolsky, 2000; Liston et al., 2006). In contrast, extinction increases the magnitude of vHipp-mPFC–evoked potentials (Hugues and Garcia, 2007). Extinction also reverses stress-induced attenuation of local field potentials evoked in the mPFC by stimulation of the afferent input from the mediodorsal thalamus (MDT) (Fucich et al., 2018). Further, input from the vHipp to the mPFC appears to modulate mPFC response to the MDT afferent, as preventing potentiation of the vHipp-to-mPFC pathway using low-frequency stimulation dampens extinction memory and prevents the extinction-associated potentiation of responses evoked in the mPFC by input from the MDT (Hugues et al., 2006; Garcia et al., 2008). Thus, silencing vHipp terminals in the IL may prevent extinction from counteracting the effects of chronic stress on both morphology of neurons in the mPFC as well as afferent-evoked responsivity of the mPFC, attenuating the beneficial behavioral effects observed 24 hours after extinction.

We have shown that extinction induces phosphorylation of the BDNF receptor TrkB in the IL (Paredes et al., 2021). In the current experiment, chemogenetically inhibiting vHipp terminals during extinction decreased the phosphorylation of TrkB. This result suggests that ventral hippocampal input to the IL during extinction plays a role in the induction of BDNF signaling in the IL. This finding aligns with work by Rosas-Vidal et al. (Rosas-Vidal et al., 2018) demonstrating that extinction of avoidance increased BDNF expression in ventral hippocampal neurons that project to the IL. Phosphorylation of the TrkB receptor at the Y515 site results in the initiation of signaling pathways associated with neuronal plasticity and protein translation, such as the mitogen-activated protein kinase-extracellular regulated protein kinase (Erk) signaling pathway. We previously reported that extinction induced phosphorylation of Erk in the IL, and Erk signaling in the IL is necessary for extinction to reverse stress-induced deficits in set shifting performance (Paredes et al., 2021). Thus, hindering BDNF signaling via chemogenetic inhibition of ventral hippocampal terminals in the IL may hinder protein translation and subsequent plasticity-associated processes necessary for the restorative effects of extinction in the IL.

Chemogenetically activating vHipp terminals in the IL in lieu of extinction treatment was sufficient to reverse stress-induced impairments in set-shifting performance tested 24 hours later. This observation aligns with studies showing the antidepressant-like effects of activating the vHipp-mPFC pathway (Carreno et al., 2016) and with our previous work demonstrating reversal of stress-induced cognitive deficits following high-frequency stimulation in the vHipp-mPFC pathway (Jett et al., 2015). Chemogenetically increasing vHipp drive to the IL may result in potentiation of this pathway, and this may be a mechanism through which vHipp activation restores IL function in stress-compromised animals. Indeed, modulating pyramidal cell activity with Gq-DREADDs results in cell depolarization, burst firing, and release of intracellular calcium stores (Alexander et al., 2009). Further, chemogenetic activation of pyramidal neurons in the mPFC restores dysfunctional glutamatergic signaling (Wang et al., 2018). It is possible that the reversal of stress-induced deficits we observed is due to vHipp terminals releasing BDNF into the IL in response to depolarization following CNO injection. However, although our results show the necessity of BDNF signaling in the IL for the beneficial effects of vHipp activation, they do not identify the source of BDNF release. Our approach cannot differentiate whether BDNF is being released by vHipp terminals or if it is released by IL neurons responding to vHipp excitatory input.

Our work highlights the crucial role of activity in the vHipp-IL pathway during extinction that is necessary to counteract the detrimental effects of stress. Extinction learning is a complex process involving distinct brain regions working in concert (Quirk et al., 2006). Regions such as the MDT and amygdala undergo extinction-associated plasticity. We have shown that optogenetically potentiating the MDT input to the mPFC reverses stress-induced deficits in set shifting, and extinction induces LTP in the MDT-mPFC pathway (Hugues et al., 2006). Future work will investigate mechanisms by which distinct afferents to the IL interact with and modulate each other, and the role of these processes in the therapeutic effects of extinction. This research may identify novel targets for pharmacologic interventions that may enhance the therapeutic efficacy of extinction learning, most notably in the context of cognitive–behavioral therapies such as exposure therapy for PTSD.

## Data Availability

The data underlying this article will be shared on reasonable request to the corresponding author.

## Interest Statement

Ms Paredes has no competing interests. Dr Morilak receives in-kind research support from Lundbeck that has no relation to the work presented in this paper.
